# Effects of dietary methyl sulfonyl methane and selenium on laying performance, egg quality, gut health indicators, and antioxidant capacity of laying hens

**DOI:** 10.5713/ab.21.0564

**Published:** 2022-04-29

**Authors:** Yoo Bhin Kim, Sang Hyeok Lee, Da-Hye Kim, Kyung-Woo Lee

**Affiliations:** 1 Department of Animal Science and Technology, Sanghuh College of Life Sciences, Konkuk University, Seoul 05029, Korea

**Keywords:** Antioxidant Capacity, Laying Hen, Laying Performance, Methyl Sulfonyl Methane, Selenium

## Abstract

**Objective:**

This study investigated the effects of dietary methyl sulfonyl methane (MSM) and selenium (Se) on the laying performance, egg quality, gut health indicators, egg yolk Se content, and antioxidant markers in laying hens.

**Methods:**

One hundred ninety-two 73-wk-old laying hens were randomly divided into four groups with eight replicates of six hens each. Four diets were prepared in a 2×2 factorial arrangement with or without MSM and Se. The trial lasted for 12 wk.

**Results:**

There were no interaction effects or main effects (p>0.05) on laying performance and egg quality. However, feed intake increased in Se-fed hens (p = 0.051) and decreased in MSM-fed hens (p = 0.067) compared with that of hens in the control group. Dietary MSM increased (p<0.05) the ileal villus height and villus height:crypt ratio in hens compared with those receiving the non-supplemented control diet. Dietary MSM and Se did not affect the percentage of short-chain fatty acids in the ileal contents. Dietary Se enriched the Se content in egg yolk compared with that of the non-supplemented control diet (p<0.05). Dietary Se increased (p<0.05) glutathione peroxidase levels in the liver and serum samples compared to the control diet. The total antioxidant capacity in the liver increased (p<0.05) in laying hens that were fed MSM-supplemented diets than in hens fed the control diet. Dietary MSM significantly increased the relative superoxide dismutase levels in serum samples (p<0.05).

**Conclusion:**

Supplementation with either MSM or Se independently improved the antioxidant capacity of laying hens. Furthermore, dietary Se produced Se-enriched eggs, but this effect was neither additive nor synergistic with dietary MSM.

## INTRODUCTION

Antioxidants play important roles in maintaining the health and performance of laying hens [1] by protecting the host from the detrimental effects of free radicals and toxic products of lipid peroxidation [2]. Thus, it has been common practice to add synthetic or natural antioxidants into the diets of chickens to alleviate oxidative stress during production [3,4].

Methyl sulfonyl methane (MSM) is a small sulfur (S)-based molecule with two double- bonded oxygen atoms and two methyl groups [5]. It is a white crystalline powder containing 34% S on a weight basis. MSM, a natural source of organic S, is a safer source of sulfate that can ameliorate oxidative stress [6]. In addition, MSM per se acts as free radical scavenger which led to an increasing interest for its potential use as the antioxidant [7]. Indeed, MSM lowers the malondialdehyde (MDA) content in experimentally induced tissue damage models *in vivo* [8] and increases antioxidant enzyme levels [9]. MSM may also have free radical-scavenging activity in ducks [10].

Selenium (Se) is an essential trace element with very similar chemical and physical properties to S. It functions as an antioxidant and improves the performance of poultry [11]. Traditionally, sodium selenite (Na_2_SeO_3_) is the most practical inorganic Se source added to chicken diets [12]. The oxidative stability of yolk lipids was improved in hens fed diets enriched with sodium selenite [13]. In addition, Se-enriched eggs may be marketed as functional foods to increase human Se intake [14].

Both S and Se are generally recognized as minerals needed in the diets of animals. They are members of Group 16 of the periodic table and share chemical and physical properties [15]. They also share biological properties, as both occur in proteins as components of amino acids such as cysteine, methionine, selenocysteine, and selenomethionine [16]. Thus, they perform important functions including as antioxidants inside the body. Although both MSM and Se act as antioxidants, their additive or synergistic effects on the antioxidant capacity of laying hens have not been evaluated. Here, we investigated the effects of dietary MSM or Se on the laying performance, egg quality, gut health indicators, and antioxidant capacity of laying hens. In addition, based on previously found [7] potentials of MSM and Se to improve gut microbiota, we monitored ileal morphology and ileal short-chain fatty acids (SCFAs) in ileal digesta. Finally, owing to the possible nutritional relationship and interaction between S and Se in animals [17], we analyzed Se content in yolk samples in the absence and presence of dietary MSM.

## MATERIALS AND METHODS

### Animal care

All experimental protocols and the use of laying hens in the trial were approved by the Institutional Animal Care and Use Committee of Konkuk University (KU20030), Republic of Korea.

### Birds and experimental design

One hundred ninety-two 73-wk-old laying hens (Lohmann Brown-Lite) with 2,036.9±20.3 g of initial body weight were randomly assigned to four experimental groups. Each group consisted of three cages housing two birds per cage (n = 6 birds/replicate; 8 replicates/treatment). The dimensions of the cage were 45 cm×45 cm×45 cm. Layers were fed one of four diets for 12 wk, namely a control diet (Table 1) and the diets containing MSM (Sigma-Aldrich, St. Louis, MO, USA) at 2 g/kg of diet, Se as sodium selenite (Innoba Tech Co., Jeongeup, Korea) at 1 mg Se/kg of diet, or both. The added levels of MSM [18] and Se [19,20] were based on previous studies showing their positive effects on mitigating oxidative stress or augmenting antioxidant capacity in chickens. The MSM contents were 0.03%, and 0.17% in the control and MSM-supplemented diets, respectively. The Se contents were 0.15, 0.22, 1.30, and 1.15 mg/kg in the control, MSM, Se, and MSM+Se diets, respectively. Feed and water were provided *ad libitum*. A lighting program of 16 h of light and 8 h of dark was used throughout the experimental period.

### Laying performance and egg quality

Feed intake per replicate was recorded and used to calculate the daily feed intake per bird. Egg production and egg weight were recorded daily and used to calculate the egg mass. The percentage of dirty and broken eggs was calculated as (total dirty and broken eggs per replicate/total eggs per replicate) ×100. The feed conversion ratio was calculated as the feed intake/egg mass per replicate.

On the last three consecutive days at 4, 8, and 12 wk, six intact eggs per replicate were collected for egg quality assessment. The eggshell color was estimated using a shell color reflectometer (TSS QCR, Technical Services and Supplies, York, UK). The Haugh unit, eggshell strength, eggshell thickness (without shell membrane) and yolk color score were assessed using a digital egg tester (DET-6000; Nabel, Kyoto, Japan). The separated yolks were weighed after clearing the adherent albumin residues with filter paper [21]. Eggshells were cleaned to remove the adherent albumen, dried at room temperature for 3 d, and weighed. The albumen weight was calculated by subtracting the yolk and dry eggshell weights from the initial egg weight.

### Egg yolk Se analysis

Three eggs per replicate sampled at 12 wk were pooled for Se determination in egg yolks. Pooled egg yolks were homogenized, frozen immediately at −20°C, and lyophilized (Labtech Freeze Dryer; Daihan, Namyangju, Korea) at −80°C. After lyophilization, the samples were ground for Se analysis. Approximately 0.5 g of sample was digested with 2.5 mL of HNO_3_ in a microwave digestion system (Multiwave 7000; Anton Paar, Graz, Austria), and the Se content of the wet-digested sample was determined by inductively coupled plasma-optical emission spectrometry (Avio 200; Perkin Elmer, Waltham, MA, USA) [22].

### Tissue, blood and digesta samplings

At 4 wk and 8 wk, one hen per replicate was randomly selected for blood sampling from the brachial vein. At 12 wk, one hen per replicate was euthanized by gradual overdose of carbon dioxide, as recommended by the ethical committee, followed by blood sampling by heart puncture. Immediately after blood sampling, the liver and digestive tract were excised. The liver was stored at −20°C until use. Ileal digesta were obtained by gently stripping the distal ileum and stored on ice until preparation on the day of sampling. For histology, a 1 cm long mid-ileal segment was fixed in 10% neutral buffered formalin for 48 h. Serum samples were obtained by gentle centrifugation (200×*g*) for 15 min and stored at −20°C before analysis [23].

### Ileal morphology

Histological sections (5 μm thick) were prepared using standard hematoxylin-eosin staining [24]. The mucosa was examined under a light microscope (Olympus BX43, Tokyo, Japan) and photographed using a digital camera (eXcope T500; DIXI Science, Daejeon, Korea). Ten intact well-oriented villi and crypts were counted for villus height and crypt depth. Villus height was measured from the villus tip to the villus bottom and crypt depth was measured from the villus bottom to the crypt. The ratio of villus height to crypt depth was then calculated.

### Analysis of short-chain fatty acids in ileal digesta

Approximately 1 g of ileal digesta was thoroughly homogenized in 4 mL of ice-cold distilled water. The homogenate was then mixed with 0.05 mL of saturated HgCl_2_, 1.00 mL of 25% H_3_PO_4_, and 0.20 mL of 2% pivalic acid and centrifuged at 1,000×*g* at 4°C for 20 min. One milliliter of supernatant was used to measure the SCFA concentration by gas chromatography (6890 Series GC System; HP, Palo Alto, CA, USA), as described by Kim et al [25].

### Antioxidant markers in serum and liver samples

A frozen liver obtained at 12 wk was thawed, and approximately 1 g of liver was homogenized (Digital Ultra-Turrax T25; IKA, Staufen, Germany) with 9 mL of cold 1×phosphate buffered saline [25]. The homogenate was then centrifuged at 10,000×*g* for 10 min, and the aliquot of the supernatant was stored at −20°C until analysis. The diluted aliquot was used to determine glutathione peroxidase (GPX) (EnzyChrom GPx; BioAssay Systems, Hayward, CA, USA) level, total antioxidant capacity (TAC) (QuantiChrom Antioxidant, BioAssay Systems, USA), catalase (CAT) (Cell Biolabs, Inc., San Diego, CA, USA) level, and MDA (Cell Biolabs, Inc., USA) according to the manufacturer’s instructions. The results were normalized against the total protein concentration in each sample. The total protein concentration in the liver was quantified using the Bradford method (Sigma, St. Louis, MO, USA).

Serum samples at 4, 8, and 12 wk were used to measure the levels of various biomarkers of oxidative stress, including GPX, superoxide dismutase (SOD), TAC, CAT, MDA, and 8-hydroxydeoxyguanosine (8-OHdG). SOD activity was analyzed using the SOD determination assay kit-WST (Sigma, USA) and expressed as SOD activity (inhibition rate %). 8-OHdG, an indicator of oxidative DNA damage, was determined using an 8-OHdG DNA Damage ELISA Kit (Cell Biolabs, Inc., USA). All assays were conducted according to the manufacture’s recommendations.

### Statistical analysis

Three adjacent cages were used as the experimental unit. Data for all variables were analyzed by a two-way analysis of variance with the model including MSM and Se as the main factors. Their interaction was determined using the general linear model procedure of SAS 9.4 [26]. There was no interaction between dietary MSM and Se. Thus, data are presented as overall means for each factor. Significant differences among treatments were determined at p<0.05.

## RESULTS

### Laying performance and egg quality

No significant interaction between MSM and Se was found with regard to laying performance (Table 2) or egg quality (Table 3). Either MSM or Se failed to affect egg production, egg weight, egg mass, feed conversion ratio, or dirty and cracked eggs at all ages. Although not statistically significant, dietary Se tended to increase (p = 0.051) the feed intake, whereas MSM tended to decrease (p = 0.067) the feed intake compared with that of laying hens fed the non-supplemented control diet. Egg components and egg quality were not affected by dietary MSM or Se supplementation. Se-fed laying hens laid 2.4% (p>0.05) thinner eggshells on average compared with the control diet-fed counterparts.

### Se deposition in egg yolk

Dietary Se increased (p<0.05) the Se content by 28.7% (3.00 mg Se/kg vs 3.86 mg Se/kg of egg yolk) in egg yolk at 12 wk irrespective of the presence of dietary MSM (Table 4).

### Ileal morphology and short-chain fatty acid concentration

No interaction between MSM and Se affected the ileal morphology (Table 5) or SCFA concentration (Table 6). Dietary MSM increased (p<0.05) both the villus height and villus height:crypt depth ratio compared with the non-supplemented control diet. However, dietary Se did not affect the ileal morphology. Neither MSM nor Se affected the percentage of SCFAs in the ileal digesta.

### Antioxidant markers in liver samples

Dietary Se, but not MSM, elevated the GPX activity in the liver compared with the non-supplemented control group (Table 7). The TAC in liver samples was increased in laying hens fed dietary MSM (p = 0.002) and Se (p = 0.067) compared with that of the hens fed the non-supplemented control. The CAT activity ranged from 57.25 U/mg to 60.41 U/mg of protein but was not affected by dietary treatments. Dietary MSM, but not Se, decreased the MDA concentration (p<0.05) compared with the non-supplemented control. No interaction effect between MSM and Se on antioxidant markers in the liver samples was detected (p>0.05).

### Antioxidant markers in serum samples

None of the treatments affected the antioxidant markers in the serum samples at 4 wk and 8 wk (data not shown). Dietary Se increased the GPX activity compared with the control group at 12 wk (Table 8). Dietary MSM, but not Se, increased the SOD activity (p = 0.014) and TAC activity (p = 0.068) in serum samples at 12 wk. However, CAT, MDA, and 8-OHdG levels were not affected by dietary MSM or Se (p>0.05).

## DISCUSSION

We found no significant effects of dietary MSM and Se on laying performance and egg quality in laying hens. The lack of effect of dietary MSM on performance has been reported in laying hens [27] and broiler chickens [28,29]. In addition, Chantiratikul et al [20] reported that dietary Se (1 mg/kg of dietary sodium selenite) also failed to affect the performance of laying hens. However, no explanation is available regarding how dietary MSM increased feed intake while dietary Se partially decreased feed intake compared with that of the control group. Whether MSM or Se has contradictory effects on the regulation of voluntary feed intake in laying hens needs to be addressed in further studies.

Egg quality is affected by genetic factors, diet, health, and environment [30]. However, dietary treatments here did not affect egg quality, which is in accordance with previous reports [14,22,30]. However, Se reduced the eggshell thickness. Arpášová et al [31] reported a decrease in eggshell strength of quails fed Se-enriched diets and noted an Se-mediated alteration in the concentrations of microelements in eggs. Nonetheless, MSM and Se did not affect eggshell weight or eggshell strength in this study.

Intestinal development can be evaluated by measuring the crypt, the region in which new intestinal cells are developed, as well as the height and surface area of the villus to determine the area available for digestion and absorption [32]. Increased villi height and decreased crypt depth can allow a greater surface area for nutrient absorption, thereby leading to improved growth performance [33]. In this study, MSM vs Se was found to be more effective in increasing the villus height and the villus height:crypt depth ratio. Our study is in line with an earlier finding [34] showing that duodenal villus height increased in broilers fed an S-supplemented diet. However, a clear explanation of the MSM-mediated improvement in ileal morphology is not available. In earlier studies, MSM was shown to exhibit antimicrobial activity or immune-modulating activity [27,35]. However, the finding that dietary MSM did not affect ileal SCFAs in this study suggests that the antimicrobial activity of MSM might not be responsible for MSM-mediated improvement in ileal morphology. Thus, further studies are warranted to investigate MSM-directed alteration in the gut microbiota or gut epithelial barrier functions.

Dietary Se can enhance the Se concentration of eggs [36]. Dietary Se leads to the incorporation of Se into egg yolk, albumen, and eggshell [30]. We also found that dietary Se was effectively transferred into the egg yolk, which agrees with previous observations [2,12,19,22]. However, our study showed that the incorporation of Se in the yolk was independent of the presence of dietary MSM.

In studies with laying hens [27] and broiler chickens [18], dietary MSM has been systemically incorporated upon ingestion. The latter studies [18], coupled with the current finding, suggest systemic availability of MSM and Se upon ingestion, thus exhibiting their biological activity in the host including antioxidant capacity. Selenium has a special function in antioxidant control mechanisms as an essential component of the active center of selenoenzymes [37]. This function helps to maintain membrane integrity and to reduce the likelihood of propagation to further oxidative damage to biomolecules such as lipids [38]. Indeed, we found that the antioxidant activities of dietary Se and MSM were noted in laying hens by either increasing GPX activity and the levels of TAC and SOD or lowering MDA in laying hens.

GPX is an Se-dependent enzyme that catalyzes the reduction of hydrogen peroxide and organic peroxides to water and the corresponding stable alcohols, respectively, thereby inhibiting the formation of free radicals [39]. We noted that the GPX activity in liver and serum samples was elevated in laying hens fed Se-enriched diets. The linear correlation between the Se content and GPX activity in blood and tissue samples has been well demonstrated [40]. Earlier studies have also shown that dietary Se increase the GPX activity in serum [19,30,41], plasma [20], and liver [2,16] samples in laying hens, which corroborates our findings.

TAC is the capacity of antioxidants required to reduce oxidants, and its measurement provides useful information about the overall antioxidant status [42]. In this study, dietary MSM increased the TAC in liver and serum samples, which agrees with a previous study on ducks exhibiting an MSM-mediated impact on higher TAC in serum [10]. In addition, we found that the Se-supplemented group had, on average, 5.5% elevated TAC in the liver compared with that in the control group, thereby confirming the antioxidant property as described by Reshadi et al [43].

Yan et al [10] indicated that low MDA levels may imply the potent antioxidant activity of dietary MSM in scavenging free radicals. The MDA levels in liver samples, but not in serum samples, were lower in laying hens fed diets containing dietary MSM. Abdul Rasheed et al [18] reported that dietary MSM was beneficial in reducing the MDA levels in the plasma and liver of broiler chickens subjected to diet-induced oxidative stress. As seen in this study, previous studies found that Se supplementation had no effect on the MDA levels in the blood and liver of laying hens [16,19,30].

SOD is an essential antioxidant enzyme defense system that catalyzes the dismutation of superoxide anions to hydrogen peroxide [44]. In this study, the supplementation of MSM increased the SOD level in serum samples by an average of 22.0% at 12 wk, which was consistent with the finding of a previous study on MSM-fed ducks [10].

In conclusion, dietary MSM or Se had no interactive effect on the laying performance and egg quality in laying hens. MSM, but not Se, improved the ileal morphology. Finally, both MSM and Se were effective in increasing the antioxidant capacity in the liver and serum of laying hens, but their antioxidant effects were independent.

## Figures and Tables

**Table 1:** Ingredient and nutrient composition of the basal diet

Items	
Ingredients	Percentage (g/100 g) of diet
Corn	41.00
Soybean meal (45% crude protein)	10.41
Wheat	12.80
Animal fat	1.02
Rice bran	2.00
Corn steep liquor	1.00
Rapeseed meal	3.00
Dried distiller’s grains with solubles	12.83
Molasses	2.00
Liquid choline (50%)	0.06
Limestone	10.51
Monodicalcium phosphate	1.02
NaCl	0.24
Variable	1.59
Methionine (99%)	0.07
Lysine (54%)	0.10
Tryptophan (10%)	0.10
Mineral mix^1)^	0.14
Vitamin mix^2)^	0.12
Total	100.00
Nutrient composition (g/100 g)	
Nitrogen-corrected apparent metabolizable energy (kcal/kg)	2,600
Dry matter^3)^	88.20
Crude protein^3)^	14.49
Crude fat^3)^	4.01
Crude ash^3)^	14.84
Calcium^4)^	4.10
Sulfur^3)^	0.15
Available phosphorus^4)^	0.28
Lysine^4)^	0.65
Methionine^4)^	0.32
Methionine+cysteine^4)^	0.60
Methyl sulfonyl methane^3)^	0.03

1) Mineral mixture provided the following nutrients per kg of diet: Fe, 50 mg; Cu, 24 mg; Zn, 90 mg; Mn, 96 mg; I, 12 mg.

2) Vitamin mixture provided following nutrients per kg of diet: vitamin A, 15,400 IU; vitamin D_3_, 3,080 IU; vitamin E, 14.000 mg; vitamin K_3_, 1.400 mg; vitamin B_1_, 1.120 mg; vitamin B_2_, 2.800 mg; vitamin B_6_, 3.920 mg; vitamin B_12_, 0.014 mg.

3) Analyzed value.

4) Calculated value.

**Table 2:** Effects of dietary methyl sulfonyl methane and selenium on laying performance of laying hens

Items	Se^1)^		MSM^2)^		SEM	p-value		
		
	−	+	−	+		Se	MSM	Se×MSM
Feed intake (g/bird)	105.37	107.79	107.71	105.45	1.07	0.051	0.067	0.443
Egg production (%)	82.53	81.59	82.57	81.54	2.56	0.739	0.716	0.778
Egg weight (g/egg)	63.35	63.35	63.68	63.43	0.76	0.638	0.762	0.559
Egg mass (g/d)	52.28	51.93	52.50	51.70	1.49	0.831	0.631	0.919
Feed conversion ratio (kg/kg)	2.03	2.09	2.07	2.05	0.05	0.281	0.816	0.744
Dirty and cracked egg (%/total)	1.86	1.73	1.75	1.84	0.24	0.683	0.757	0.701

SEM, standard error of the means.

1) −, selenium at 0 mg/kg of diet; +, selenium at 1 mg/kg of diet.

2) 0, methyl sulfonyl methane at 0 g/kg of diet; 0.2, methyl sulfonyl methane at 2 g/kg of diet.

**Table 3:** Effects of dietary methyl sulfonyl methane and selenium on egg composition and egg quality of laying hens

Items	Se^1)^		MSM^2)^		SEM	p-value		
		
	−	+	−	+		Se	MSM	Se×MSM
Relative yolk weight (%)	25.99	26.40	26.25	26.15	0.32	0.217	0.761	0.225
Relative albumen weight (%)	64.11	63.71	63.85	63.97	0.33	0.261	0.732	0.223
Relative eggshell weight (%)	9.90	9.87	9.89	9.88	0.09	0.708	0.960	0.927
Yolk color	6.51	6.57	6.56	6.52	0.05	0.241	0.468	0.625
Haugh unit	73.74	72.57	72.61	73.71	0.67	0.168	0.191	0.768
Eggshell strength (kg/cm^2^)	4.60	4.60	4.60	4.61	0.08	0.993	0.918	0.967
Eggshell thickness (mm)	0.42	0.41	0.41	0.41	0.00	0.052	0.956	0.807
Eggshell color unit	27.92	27.89	27.89	27.92	0.45	0.960	0.953	0.815

SEM, standard error of the means.

1) −, selenium at 0 mg/kg of diet; +, selenium at 1 mg/kg of diet.

2) 0, methyl sulfonyl methane at 0 g/kg of diet; 0.2, methyl sulfonyl methane at 2 g/kg of diet.

**Table 4:** Effects of dietary methyl sulfonyl methane and selenium on selenium content in egg yolks

Treatment		Se (mg/kg)
Se^1)^	−	3.00^b^
	+	3.86^a^
MSM^2)^	−	3.39
	+	3.47
SEM		0.12
p-value		
Se		<0.001
MSM		0.546
Se×MSM		0.636

SEM, standard error of the means.

1) −, selenium at 0 mg/kg of diet; +, selenium at 1 mg/kg of diet.

2) 0, methyl sulfonyl methane at 0 g/kg of diet; 0.2, methyl sulfonyl methane at 2 g/kg of diet.

a,b Values without a common superscript letter within a column differ significantly (p<0.05).

**Table 5:** Effects of dietary methyl sulfonyl methane and selenium on ileal morphology in laying hens

Items	Se^1)^		MSM^2)^		SEM	p-value		
		
	−	+	−	+		Se	MSM	Se×MSM
Villus height (μm)	888.59	921.05	860.58^b^	949.07^a^	35.49	0.374	0.024	0.409
Crypt depth (μm)	128.23	126.92	131.54	123.61	7.14	0.857	0.283	0.963
Villus height:crypt depth ratio	6.99	7.33	6.60^b^	7.72^a^	0.35	0.342	0.006	0.526

SEM, standard error of the means.

1) −, selenium at 0 mg/kg of diet; +, selenium at 1 mg/kg of diet.

2) 0, methyl sulfonyl methane at 0 g/kg of diet; 0.2, methyl sulfonyl methane at 2 g/kg of diet.

a,b Values without a common superscript letter within a same row differ significantly (p<0.05).

**Table 6:** Effects of dietary methyl sulfonyl methane and selenium on the percentages of ileal short-chain fatty acids in laying hens

Items	Se^1)^		MSM^2)^		SEM	p-value		
		
	−	+	−	+		Se	MSM	Se×MSM
Acetate	70.80	70.00	69.43	71.37	3.90	0.843	0.632	0.877
Propionate	6.86	7.75	7.16	7.44	1.15	0.458	0.810	0.629
Isobutyrate	5.34	6.20	5.39	6.15	1.29	0.524	0.573	0.807
Butyrate	7.18	5.93	7.11	6.01	0.92	0.196	0.256	0.137
Isovalerate	4.86	4.82	5.00	4.67	0.64	0.949	0.617	0.378
Valerate	4.97	5.32	5.92	4.36	0.80	0.674	0.074	0.563

SEM, standard error of the means.

1) −, selenium at 0 mg/kg of diet; +, selenium at 1 mg/kg of diet.

2) 0, methyl sulfonyl methane at 0 g/kg of diet; 0.2, methyl sulfonyl methane at 2 g/kg of diet.

**Table 7:** Effects of dietary methyl sulfonyl methane and selenium on liver oxidative stress markers in laying hens

Items	Se^1)^		MSM^2)^		SEM	p-value		
		
	−	+	−	+		Se	MSM	Se×MSM
GPX activity (U/mg protein)	54.92^b^	69.59^a^	62.54	61.96	3.29	<0.001	0.199	0.777
TAC (nmol/mg protein)	58.75	61.97	57.26^b^	63.45^a^	1.48	0.069	0.002	0.058
CAT (U/mg protein)	58.47	59.58	58.83	59.22	3.83	0.777	0.920	0.599
MDA (nmol/mg protein)	2.08	2.13	2.36^a^	1.85^b^	0.22	0.825	0.026	0.721

SEM, standard error of the means; GPX, glutathione peroxidase; TAC, total antioxidant capacity; CAT, catalase; MDA, malondialdehyde.

1) −, selenium at 0 mg/kg of diet; +, selenium at 1 mg/kg of diet.

2) 0, methyl sulfonyl methane at 0 g/kg of diet; 0.2, methyl sulfonyl methane at 2 g/kg of diet.

a,b Values without a common superscript letter within a same row differ significantly (p<0.05).

**Table 8:** Effects of dietary MSM and Se on serum antioxidant markers in laying hens at 12 wk

Items	Se^1)^		MSM^2)^		SEM	p-value		
		
	−	+	−	+		Se	MSM	Se×MSM
GPX activity (U/L)	564.31^b^	750.37^a^	646.81	667.87	35.62	0.001	0.644	0.896
SOD activity (%)	90.30	91.18	81.74^b^	99.74^a^	6.59	0.897	0.014	0.251
TAC (mM)	1.41	1.53	1.34	1.59	0.12	0.351	0.068	0.740
CAT (U/mL)	2.48	2.26	2.35	2.39	0.20	0.311	0.861	0.868
MDA (μM)	28.21	29.03	31.31	25.93	3.88	0.879	0.324	0.888
8-OHdG (ng/mL)	2.27	2.40	2.41	2.26	0.73	0.896	0.886	0.900

SEM, standard error of the means; GPX, glutathione peroxidase; SOD, superoxide dismutase; TAC, total antioxidant capacity; CAT, catalase; MDA, malondialdehyde; 8-OHdG, 8-hydroxydeoxyguanosine.

1) −, selenium at 0 mg/kg of diet; +, selenium at 1 mg/kg of diet.

2) 0, methyl sulfonyl methane at 0 g/kg of diet; 0.2, methyl sulfonyl methane at 2 g/kg of diet.

a,b Values without a common superscript letter within a row differ significantly (p<0.05).

## References

[1] Surai PF, Kochish II, Romanov MN, Griffin DK (2019). Nutritional modulation of the antioxidant capacities in poultry: the case of vitamin E. Poult Sci.

[2] Surai PF (2020). Antioxidants in poultry nutrition and reproduction: An update. Antioxidants.

[3] Karre L, Lopez K, Getty KJK (2013). Natural antioxidants in meat and poultry products. Meat Sci.

[4] Edrees GM, Serag HM, EL-Gogary MR, Alsharif AA (2017). Effect of natural antioxidants supplementation as feed ingredients in laying hen diet. J Anim Poult Prod.

[5] Withee ED, Tippens KM, Dehen R, Tibbitts D, Hanes D, Zwickey H (2017). Effects of Methylsulfonylmethane (MSM) on exercise-induced oxidative stress, muscle damage, and pain following a half-marathon: A double-blind, randomized, placebo-controlled trial. J Int Soc Sports Nutr.

[6] Kim YH, Kim DH, Lim H, Baek DY, Shin HK, Kim JK (2009). The anti-inflammatory effects of methylsulfonylmethane on lipopolysaccharide-induced inflammatory responses in murine macrophages. Biol Pharm Bull.

[7] Butawan M, Benjamin RL, Bloomer RJ (2017). Methylsulfonylmethane: Applications and safety of a novel dietary supplement. Nutrients.

[8] Kamel R, El Morsy EM (2013). Hepatoprotective effect of methylsulfonylmethane against carbon tetrachloride-induced acute liver injury in rats. Arch Pharm Res.

[9] Amirshahrokhi K, Bohlooli S (2013). Effect of methylsulfonylmethane on paraquat-induced acute lung and liver injury in mice. Inflammation.

[10] Yan HL, Cao SC, Hu YD, Zhang HF, Liu JB (2020). Effects of methylsulfonylmethane on growth performance, immunity, antioxidant capacity, and meat quality in Pekin ducks. Poult Sci.

[11] Mechora Š, Žerdoner Čalasan A, Felicijan M, Urbanek Krajnc A, Ambrožič-Dolinšek J (2017). The impact of selenium treatment on some physiological and antioxidant properties of Apium repens. Aquat Bot.

[12] Jing CL, Dong XF, Wang ZM, Liu S, Tong JM (2015). Comparative study of DL-selenomethionine vs sodium selenite and seleno-yeast on antioxidant activity and selenium status in laying hens. Poult Sci.

[13] Skřivan M, Marounek M, Englmaierová M, Skřivanová V (2013). Influence of dietary vitamin C and selenium, alone and in combination, on the performance of laying hens and quality of eggs. Czech J Anim Sci.

[14] Muhammad AI, Mohamed DA, Chwen LT, Akit H, Samsudin AA (2021). Effect of selenium sources on laying performance, egg quality characteristics, intestinal morphology, microbial population and digesta volatile fatty acids in laying hens. Animals.

[15] Hoffmann JE, King MG (2000). Selenium and selenium compounds. Kirk-Othmer Encycl Chem Technol.

[16] Jacob C, Giles GI, Giles NM, Sies H (2003). Sulfur and selenium: The role of oxidation state in protein structure and function. Angew Chemie - Int Ed.

[17] Ivancic J, Weiss WP (2001). Effect of dietary sulfur and selenium concentrations on selenium balance of lactating Holstein cows. J Dairy Sci.

[18] Abdul Rasheed MS, Oelschlager ML, Smith BN, Bauer LL, Whelan RA, Dilger RN (2020). Dietary methylsulfonylmethane supplementation and oxidative stress in broiler chickens. Poult Sci.

[19] Meng T, Liu Y, Xie C (2019). Effects of different selenium sources on laying performance, egg selenium concentration, and antioxidant capacity in laying hens. Biol Trace Elem Res.

[20] Chantiratikul A, Orawan C, Chantiratikul P (2008). Effect of sodium selenite and zinc-L-selenomethionine on performance and selenium concentrations in eggs of laying hens. Asian-Australas J Anim Sci.

[21] Lee SH, Kim YB, Kim D-H (2021). Dietary soluble flaxseed oils as a source of omega-3 polyunsaturated fatty acids for laying hens. Poult Sci.

[22] Liu H, Yu Q, Tang X, Fang C, Chen S, Fang R (2020). Effect of selenium source and level on performance, egg quality, egg selenium content, and serum biochemical parameters in laying hens. Foods.

[23] Lee JW, Kim DH, Kim YB (2020). Dietary encapsulated essential oils improve production performance of coccidiosis-vaccine-challenged broiler chickens. Animals.

[24] Kim Y, Lee S, Kim D (2022). Effects of dietary organic and inorganic sulfur on laying performance, egg quality, ileal morphology, and antioxidant capacity in laying hens. Animals.

[25] Kim YB, Kim DH, Jeong SB (2020). Black soldier fly larvae oil as an alternative fat source in broiler nutrition. Poult Sci.

[26] SAS (2012). Statistical Analyses System version 94.

[27] Lim CI, Choe HS, Kang C, Lee BK, Ryu KS (2018). Effects of dietary organic sulfur on performance, egg quality and cell-mediated immune response of laying hens. Korean J Poult Sci.

[28] Park SO, Shin JH, Choi WK, Park BS (2010). Effect of feeding dietary legislation sulfur as an antibiotic replacement in broiler chickens. Ann Anim Resour Sci.

[29] Jiao Y, Park JH, Kim YM, Kim IH (2017). Effects of dietary methyl sulfonyl methane (MSM) supplementation on growth performance, nutrient digestibility, meat quality, excreta microbiota, excreta gas emission, and blood profiles in broilers. Poult Sci.

[30] Han XJ, Qin P, Li WX (2017). Effect of sodium selenite and selenium yeast on performance, egg quality, antioxidant capacity, and selenium deposition of laying hens. Poult Sci.

[31] Arpášová H, Petrovič V, Mellen M, Kačániová M, Čobanová K, Leng L (2009). The effects of supplementing sodium selenite and selenized yeast to the diet for laying hens on the quality and mineral content of eggs. J Anim Feed Sci.

[32] Laudadio V, Passantino L, Perillo A (2012). Productive performance and histological features of intestinal mucosa of broiler chickens fed different dietary protein levels. Poult Sci.

[33] Duangnumsawang Y, Zentek J, Goodarzi Boroojeni F (2021). Development and functional properties of intestinal mucus layer in poultry. Front Immunol.

[34] Park SO, Park BS (2017). Dietary sulphur as alternative antibacterial supplements for broiler chickens. Eur Poult Sci.

[35] Poole TL, Benjamin R, Genovese KJ, Nisbet DJ (2019). Methylsulfonylmethane exhibits bacteriostatic inhibition of Escherichia coli, and Salmonella enterica Kinshasa, in vitro. J Appl Microbiol.

[36] Lu J, Qu L, Shen MM (2018). Comparison of dynamic change of egg selenium deposition after feeding sodium selenite or selenium-enriched yeast. Poult Sci.

[37] Zuberbuehler CA, Messikommer RE, Arnold MM, Forrer RS, Wenk C (2006). Effects of selenium depletion and selenium repletion by choice feeding on selenium status of young and old laying hens. Physiol Behav.

[38] Habibian M, Sadeghi G, Ghazi S, Moeini MM (2015). Selenium as a feed supplement for heat-stressed poultry: a review. Biol Trace Elem Res.

[39] Surai PF, Kochish II, Fisinin VI (2018). Glutathione peroxidases in poultry biology: Part 1. Classification and mechanisms of action. Worlds Poult Sci J.

[40] Pavlata L, Illek J, Pechová A (2001). Blood and tissue selenium concentrations in calves treated with inorganic or organic selenium compounds - a comparison. Acta Vet Brno.

[41] Liu H, Yu Q, Tang X, Fang C, Chen S, Fang R (2020). Effect of selenium on performance, egg quality, egg selenium content and serum antioxidant capacity in laying hens. Pak J Zool.

[42] Korzeniowska M, Króliczewska B, Kopeć W (2018). Effect of dietary selenium on protein and lipid oxidation and the antioxidative potential of selected chicken culinary parts during frozen storage. J Chem.

[43] Reshadi H, Torki M, Mohammadi H (2020). Changes in performance, egg quality and blood parameters of laying hens fed selenium and oregano oil. Anim Prod Sci.

[44] Gordillo Jaramillo FX, Kim DH, Lee SH, Kwon SK, Jha R, Lee KW (2021). Role of oregano and Citrus species-based essential oil preparation for the control of coccidiosis in broiler chickens. J Anim Sci Biotechnol.

